# Predominant Microbial Colonizers in the Root Endosphere and Rhizosphere of Turfgrass Systems: *Pseudomonas veronii*, *Janthinobacterium lividum*, and *Pseudogymnoascus* spp.

**DOI:** 10.3389/fmicb.2021.643904

**Published:** 2021-03-23

**Authors:** Qing Xia, Thomas Rufty, Wei Shi

**Affiliations:** Department of Crop and Soil Sciences, North Carolina State University, Raleigh, NC, United States

**Keywords:** microbiome, *Pseudomonas*, endophytes, rhizosphere, turfgrass

## Abstract

Microbes can colonize plant roots to modulate plant health and environmental fitness. Thus, using microbes to improve plant adaptation to biotic and abiotic stresses will be promising to abate the heavy reliance of management systems on synthetic chemicals and limited resource. This is particularly important for turfgrass systems because intensive management for plant available nutrients (e.g., nitrogen), water, and pest control is necessary to maintain a healthy and aesthetic landscape. However, little is known on microbial species and host compatibility in turfgrass root endosphere and rhizosphere. Here, by using marker gene high throughput sequencing approaches we demonstrated that a few bacterial and fungal species prevailed the root endosphere and rhizosphere and were of a broad host spectrum. Irrespective of turfgrass species (bermudagrass, ultradwarf bermudagrass, creeping bentgrass, and tall fescue), defoliation intensities (i.e., mowing height and frequency), turfgrass sites, and sampling time, *Pseudomonas veronii* was predominant in the root endosphere, constituting ∼38% of the total bacterial community, which was much higher than its presence in the bulk soil (∼0.5%) and rhizosphere (∼4.6%). By contrast, *Janthinobacterium lividum* and fungal species of the genus *Pseudogymnoascus* were more abundant in the rhizosphere, constituting ∼15 and ∼ 39% of the total bacterial and fungal community, respectively, compared to their respective presence in the bulk soil (∼ 0.1 and 5%) and root endosphere (∼ 0.8 and 0.3%). Such stark contrasts in the microbiome composition between the root endosphere, rhizosphere, and bulk soil were little influenced by turfgrass species, suggesting the broad turfgrass host compatibility of these bacterial and fungal species. Further, their dominance in respective niches were mutually unaffected, implying the possibility of developing a multiple species formula for coping turfgrass with environmental stresses. These species were likely involved in controlling pests, such as infectious nematodes and fungi, decomposing root debris, and helping turfgrass water and nutrient uptake; yet these possibilities need to be further examined.

## Introduction

Microbes inhabiting roots and rhizosphere can help plants to cope with biotic and abiotic stresses and thereby promote plant growth under adverse environmental conditions (Glick, 2012; Rolli et al., 2015; Timmusk et al., 2017). Mechanisms by which microbes contribute to plant nutrient uptake, stress tolerance, and defense against pathogens are multifaceted and possible ones include, but not limited to controls on phytohormone production, reactive oxygen species scavenging, rhizophagy cycle, phosphorus solubilization, osmolytic adjustment, and antibiotic synthesis (Haas and Keel, 2003; Baca and Elmerich, 2007; Sandhya et al., 2010; Ansary et al., 2012; Alori et al., 2017; White et al., 2018). This has stimulated a great interest in developing microbial inoculant-based biotechnology for making managed systems more adaptive to environmental stresses as well as more ecologically friendly.

Sustainable turfgrass management is always a challenge to the turfgrass industry as well as the society because intensive management in terms of plant nutrition, water, and disease control, is required to maintain a healthy and aesthetic landscape, e.g., urban lawns, golf courses, and recreational greens space (Strandberg et al., 2012; Ignatieva et al., 2015). Lawns have been ranked the top irrigated agricultural “crop” in the United States, over corn, wheat, and fruit orchards combined (Milesi et al., 2005). Urban and suburban landscapes also receive higher rates of pesticides than agriculture (Pimentel et al., 1993). In addition, nitrogen is also applied to turfgrass in rates comparable to grain crops, such as corn and wheat. Given the wide consensus that the plant-associated microbiome can shape the host fitness to the environment, we questioned what microbes in the turfgrass root endosphere and rhizosphere would be beneficial to sustainable turfgrass systems.

Our knowledge on turfgrass root-associated beneficial microbes is fragmented because early and sporadic investigations focused mainly on individual microbial species or taxonomic groups, particularly on fungi from cool-season grasses (Potter et al., 1992; Hyakumachi, 1994; Koske et al., 1994; Schardl and Phillips, 1997; Elliott et al., 2004). Some studies appear to support that fungal endophytes were host species specific, being less compatible in Kentucky bluegrass than in tall fescue and perennial ryegrass. Only in the recent decade, a community level approach via marker gene sequencing has been applied to examine the bacterial community in the turfgrass rhizosphere, demonstrating the predominance of some Proteobacteria, e.g., the genus *Rhodoplanes* and their potential benefits in coping turfgrass with environmental stresses (Crouch et al., 2017; Fu et al., 2020). Still, a lingering and urgent question: to what extent turfgrass species affect their microbiomes, remains unanswered. Successfully addressing this question is important for understanding the compatibility of a microbial inoculant to the host turfgrass and the associated microbiome and therefore may help harness microbes for sustainable turfgrass management.

Microbes of different taxa can co-exist in almost any habitats on earth, including plant roots, although the number of different microbial species is much less in roots than in the rhizosphere and bulk soil (Gottel et al., 2011). Differences in growing condition, survival strategy, and resource-use pattern between microbial species may require them to explore environments in a cooperative and complementary manner during some, if not all, periods of their life cycle. We hypothesized that a few microbial species might be prevalent within and outside of turfgrass roots and function in a cohort way in response to the turfgrass environment. The objective of this work, therefore, was to systematically examine bacterial and fungal communities inhabiting the root endosphere, rhizosphere and bulk soil across a range of turfgrass species/cultivars, management practices, and environmental conditions.

## Materials and Methods

### Turfgrass and Soil Sampling

Turfgrass and soil were collected during November 2018–May 2019 from different sites of mono-cultured systems in Raleigh, NC, United States (Supplementary Table 1). The four turfgrass species/cultivars were tall fescue (*Festuca arundinacea*), creeping bentgrass (*Agrostis stolonifera*), bermudagrass (*Cynodon dactylon*), and ultradwarf bermudagrass. Intact turfgrass-soil cores of creeping bentgrass, bermudagrass, and ultradwarf bermudagrass were collected from multiple sites of professionally managed golf courses, but intact cores of tall fescue were from three non-research ornamental lawns. At each individual site, six intact cores (2.5 cm dia. × 10 cm height) were randomly sampled and then pooled together as a composite sample. Thus, a total of 12 composite turfgrass-soil samples were obtained, representing four species/cultivars and three sites for each turfgrass species/cultivar. It should be noted that soil cores were stored at 4°C before composite mixing and root separation.

### Preparation of Turfgrass Root, Rhizosphere, and Bulk Soil Samples

Turfgrass root, rhizosphere, and bulk soil samples were prepared using a modified bleach-washing protocol (McPherson et al., 2018) to minimize potential risks of fine roots destruction and cross contamination associated often with a sonication method. All the glassware, spatula, and scissors used for separating roots and rhizosphere from the bulk soil were sterilized by 70% ethanol between samples to avoid contamination. Besides, all the solutions described below were sterilized by 0.22 μm filtration before use. In brief, intact grass-soil cores were destructed using a sterilized spatula. Bulk soil samples were collected from soils that were loosely associated with or unattached on grass roots and then sieved (<2 mm). Grass roots with closely attached soil were carefully separated from shoots, placed into 50 mL centrifuge tubes, and shaken for 2 min in 35 mL phosphate buffer (6.33 g/L NaH_2_PO_4_, 8.5 g/L Na_2_HPO_4_ anhydrous, 200 μL/L Silwet L-77, pH 6.5). The roots were blotted clean and washed sequentially by shaking for 60 s with 50% bleach (0.01% Tween 20), 70% ethanol, and sterilized water. Following each washing step, at least two times of additional washing with sterilized water were added to facilitate removal of solvent residuals. Root samples were blotted dry, and rhizosphere soil samples were obtained by filtration (100 μm mesh) of collected soil slurries and then centrifugation at 3,000 × *g* for 5 min. Grass root, rhizosphere, and bulk soil samples were stored at −20°C prior to DNA extraction. Aliquots of bulk soil samples were also stored at 4°C before the analysis of soil physicochemical properties.

### Soil Physicochemical Properties

Soil bulk density was estimated from the volume and mass of soil cores after deducting the mass of cobbles and stones. Soil moisture content was determined by oven drying the bulk soil at 105°C for at least 24 h. Soil pH was measured with soil slurries at a soil (g)/water (mL) ratio of 1:2.5. Soil inorganic nitrogen was quantified using a FIA QuikChem 8000 autoanalyzer (Lachat 147 Instruments, Loveland, CO, United States) after extraction with 1.0 M KCl at a soil (g)/water (mL) ratio of 1:5. Soil total C and N were analyzed by the dry combustion method with a Perkin-Elmer 2400 CHN analyzer (Perkin-Elmer Corporation, Norwalk, CT, United States). Soil physicochemical properties associated with individual sites and grass species/cultivars are given in Supplementary Table 2; and the statistic overview of soil properties is given in Table 1.

**TABLE 1:** Statistics of physicochemical properties of soil samples collected from different grass species/cultivars, defoliation intensities, sites, and growing seasons.

	Bulk density (g cm^–3^)	Moisture^*a*^ (%)	pH	Ammonium	Nitrate	Inorganic N	Total C	Total N
		
				(μg N g^−1^ soil)			(g C or N kg^−1^ soil)	
Mean	1.20	13.9	6.4	9.6	19.9	29.5	15.3	1.3
Median	1.24	12.3	6.4	8.5	9.8	21.8	15.2	1.3
Min	0.99	3.9	5.6	0.0	0.0	8.6	2.7	0.2
Max	1.37	34.4	7.5	34.2	49.1	78.7	30.3	2.6
SD	0.14	0.9	0.5	10.1	19.9	21.9	8.6	0.7
CV	11.5	67.4	8.4	108.7	103.0	76.6	58.2	61.1

*^*a*^Moisture, gravimetric moisture content; Min, minimum value; Max, maximum value; SD, standard deviation; CV, percentage of the coefficient of variation.*

### DNA Extraction and Library Preparation

Microbial genomic DNA was extracted from bulk soil (∼500 mg), rhizosphere soil (∼100–400 mg), and grass roots (∼100–200 mg) with the FastDNA Spin Kit for soil (MP Bio, Solon, OH, United States). DNA concentrations were then determined using a NanoDrop Spectrophotometer (Thermo 159 Scientific, Wilmington, DE, United States). PCR amplification of bacterial 16S rRNA genes and fungal ITS regions were performed with Illumina-overhang-added primer pairs targeting bacterial V5-V6-V7 (799F: 5′-AACMGGATTAGATACCCKG-3′ and 1193R: 5′-ACGTCATCCCCACCTTCC-3′) and fungal ITS1-ITS2 (F_KYO2: 5′-162 TAGAGGAAGTAAAAGTCGTAA-3′ and R_KYO2: 5′-TTYRCTRCGTTCTTCATC-3′) (Toju et al., 2012; Beckers et al., 2016), respectively, in a 25 μL PCR reaction with 12.5 μL KAPA HiFi HotStart ReadyMix (KAPA Biosystems, Wilmington, MA, United States), 12.5 ng template of genomic DNA, and 5 mM of each primer. The thermal conditions for PCR amplification of both bacteria and fungi were: initial denaturation at 95°C for 3 min; 30 cycles of 98°C for 30 s, 51°C for 15 s, and 72°C for 30 s, followed by a final elongation at 72°C for 5 min. The amplified PCR products were then cleaned with AMPure XP beads (Beckman Coulter Genomics, Danvers, MA, United States), and had Illumina adapters (Nextera XT index Kit, Illumina, San Diego, CA, United States) added by a second 25 μL-PCR reaction of 12.5 μL KAPA HiFi HotStart ReadyMix, 5 μL of amplified PCR products (eluted in 10 mM Tris buffer pH 8.5), and 5 mM of each primer. After a second clean-up on the PCR products, all the amplicons were diluted to 20 nM, mixed equimolarly, and paired-end sequenced (300 bp × 2) on Illumina Miseq platform (Illumina, San Diego, CA, United States). Sequence reads were deposited into the NCBI Sequence Read Archive (SRA) database under the Bioproject accession number PRJNA680169.

### Bioinformatics and Statistical Analysis

Demultiplexed sequencing reads had primers and adapters removed by cutadapt (v1.18) (Martin, 2011) and were then processed in R (3.6.1) using DADA2 (v1.12.1) pipeline (Callahan et al., 2016; R Core Team, 2020) to generate a table of amplicon sequence variants (ASVs) following dereplication, error model training, pair-end merging, and chimeras removal. Thereafter, Singletons were removed before diversity analysis and taxonomic classification in QIIME 2 (2019.7, Bolyen et al., 2019). The bacterial and fungal ASVs were annotated using Greengenes database (v13.8) and UNITE database (v8.2), respectively (DeSantis et al., 2006; Nilsson et al., 2019), and reads that were not classified as bacteria or fungi were filtered out. Microbial alpha diversity metrics (observed ASVs, Shannon index, evenness, and Chao1) were estimated, and the Bray–Curtis dissimilarity matrixes were used for both bacterial and fungal beta diversity analyses based on the sequence depth rarefied to 18,000 for 16S rRNA and 15,000 for the ITS region.

Data normality was examined by the Shapiro–Wilk test, with *P* < 0.05 for most data, indicating non-normal distribution. Therefore, the non-parametric Kruskal–Wallis test and Spearman rank correlation were used to evaluate associations of relative abundance of microbial taxonomic groups with soil physicochemical properties and turfgrass system/sampling variables. Differences in beta diversity was assessed by the non-parametric permutational multivariate analysis of variance (PERMANOVA). Associations between Bray–Cutis dissimilatory metrics and soil physicochemical properties were performed with a beta-correlation analysis in QIIME 2. To visualize how predominant ASVs in the endosphere, rhizosphere, and bulk soil overlapped, a venn diagram was made using the VennDiagram package in R (v1.6.20, Chen and Boutros, 2011). Co-occurrence patterns of predominant bacterial and fungal species were analyzed by the Spearman rank correlation and presented in an adjacency matrix using the Correlograms in R packages (corrplot v0.84, Wei and Simko, 2017).

## Results

### Microbial Diversity in the Root Endosphere, Rhizosphere, and Bulk Soil

All the four alpha diversity metrics were generally highest for the bulk soil, middle for the rhizosphere, and lowest for the root endosphere (Table 2). Compared to the bulk soil, observed and estimated ASVs for bacteria and fungi in the root endosphere were reduced by ∼70 and 77%, respectively. Evenness also declined by ∼36% for bacteria and ∼20% for fungi. Thus, Shannon index, a combined index of both species richness and evenness, declined by ∼47 and 37% for bacteria and fungi, respectively. Bacterial and fungal alpha diversity metrics in the root endosphere or rhizosphere were little associated with soil physicochemical properties or turfgrass system/sampling variables (Supplementary Figure 1), except for marginally significant correlations (*P* < 0.1) between some diversity metrics and turfgrass species/cultivars (or soil NH_4_^+^). In contrast, bacterial evenness and fungal species richness in the bulk soil were significantly (*P* < 0.05) associated with most of soil properties and system variables, such as bulk density, moisture, nitrate, total soil C and N, and turfgrass defoliation intensity.

**TABLE 2:** Bacterial and fungal alpha diversity metrics in the bulk soil, rhizosphere, and grass root endosphere of turf samples that were collected from different grass species/cultivars, defoliation intensities, sites, and growing seasons.

	ASVs	Evenness	Shannon index	Chao1
**Bacteria**				
Bulk	1513 ± 83a	0.874 ± 0.010a	9.214 ± 0.122a	1571 ± 94a
Rhizosphere	965 ± 78b	0.738 ± 0.012b	7.284 ± 0.132b	1007 ± 90b
Endosphere	448 ± 52c	0.555 ± 0.026c	4.865 ± 0.289c	490 ± 64c
**Fungi**				
Bulk	723 ± 39a	0.666 ± 0.026a	6.317 ± 0.258a	813 ± 44a
Rhizosphere	555 ± 89a	0.496 ± 0.044b	4.527 ± 0.502b	731 ± 104a
Endosphere	172 ± 19b	0.530 ± 0.027b	3.920 ± 0.259b	185 ± 20b

*Diversity metrics are expressed as means ± standard errors for *n* = 12. Different letters indicate significant differences (*P* < 0.05) between the bulk soil, rhizosphere, and root endosphere within the bacterial or fungal kingdom.*

Spearman’s rank correlations were performed for diversity metrics between the bulk soil, rhizosphere, and root endosphere to evaluate how the microbial diversity in the root endosphere was affected by diversity in the bulk soil and rhizosphere. None of the diversity metrics showed significant correlations between the root endosphere and rhizosphere, but bacterial species richness in the root endosphere was moderately related to that in the bulk soil (ρ = 0.587, *P* < 0.05 for the observed ASVs; ρ = 0.510, *P* < 0.1 for Chao1). Bacterial and fungal diversity metrics in the rhizosphere were also moderately associated to those in the bulk soil (ρ = 0.552, *P* < 0.1 for bacterial Shannon index; ρ = 0.615, *P* < 0.05 for fungal Shannon index; ρ = 0.629, *P* < 0.05 for fungal species evenness).

**FIGURE 1:**
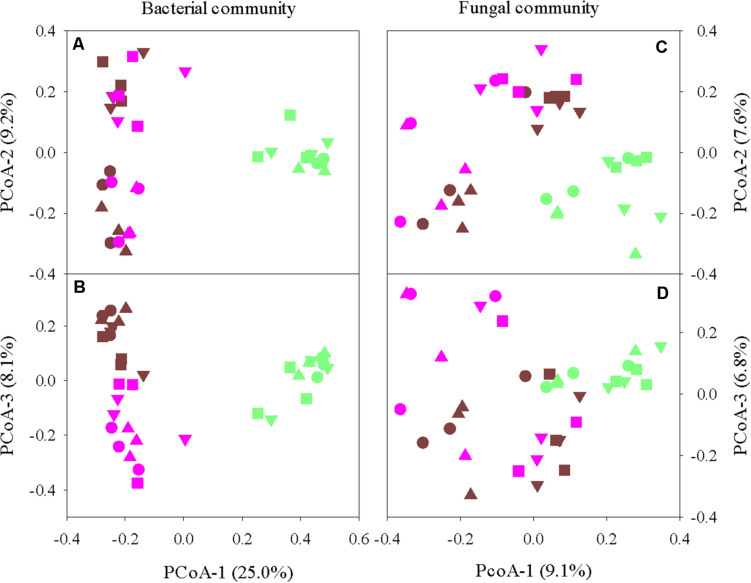
Principal coordinate analysis of bacterial **(A,B)** and fungal **(C,D)** communities in the grass root endosphere (green), rhizosphere (pink), and bulk soil (brown). Four turfgrass species/cultivars are indicted by symbols: circles, bermudagrass; squares, ultradwarf bermudagrass; upward triangle, tall fescue; and downward triangle, creeping bentgrass.

Principal coordinate analysis revealed clear differentiations in both bacterial and fungal communities among the root endosphere, rhizosphere, and bulk soil (*P* < 0.001) (Figure 1). Irrespective of turfgrass species/cultivars, defoliation intensity, sampling site and time, bacterial (or fungal) communities in the endosphere clustered together and were well separated from those in the bulk soil and rhizosphere. Bacterial (or fungal) communities also significantly differed between the bulk soil and rhizosphere, although the separation was not as clear-cut as between the endosphere and bulk soil (or rhizosphere). Such niche-based grouping was more pronounced for bacteria than for fungi, partially because system variables and soil physicochemical properties affected the fungal community more strongly and thus concealed the fungal clustering (Supplementary Figure 2). For example, fungal Bray–Curtis distance matrix was moderately and yet significantly associated with soil bulk density, total C, and total N (Spearman’s ρ = ∼0.35, *P* < 0.001), but bacterial Bray–Curtis distance matrix was unrelated to soil physicochemical properties. Also, turfgrass species, defoliation intensity, and sampling site and time all strongly affected the fungal community but little on the bacterial community. Nonetheless, the overall structures of both bacterial and fungal communities in the root endosphere were, to some degree, associated with the respective bacterial and fungal community structures in the bulk soil and rhizosphere, as shown by the robust and significant Spearman’s rank correlations between eigenvalues along individual ordination axes (Supplementary Figure 3).

### Preferential Microbial Taxa in the Root Endosphere, Rhizosphere, and Bulk Soil

**FIGURE 2:**
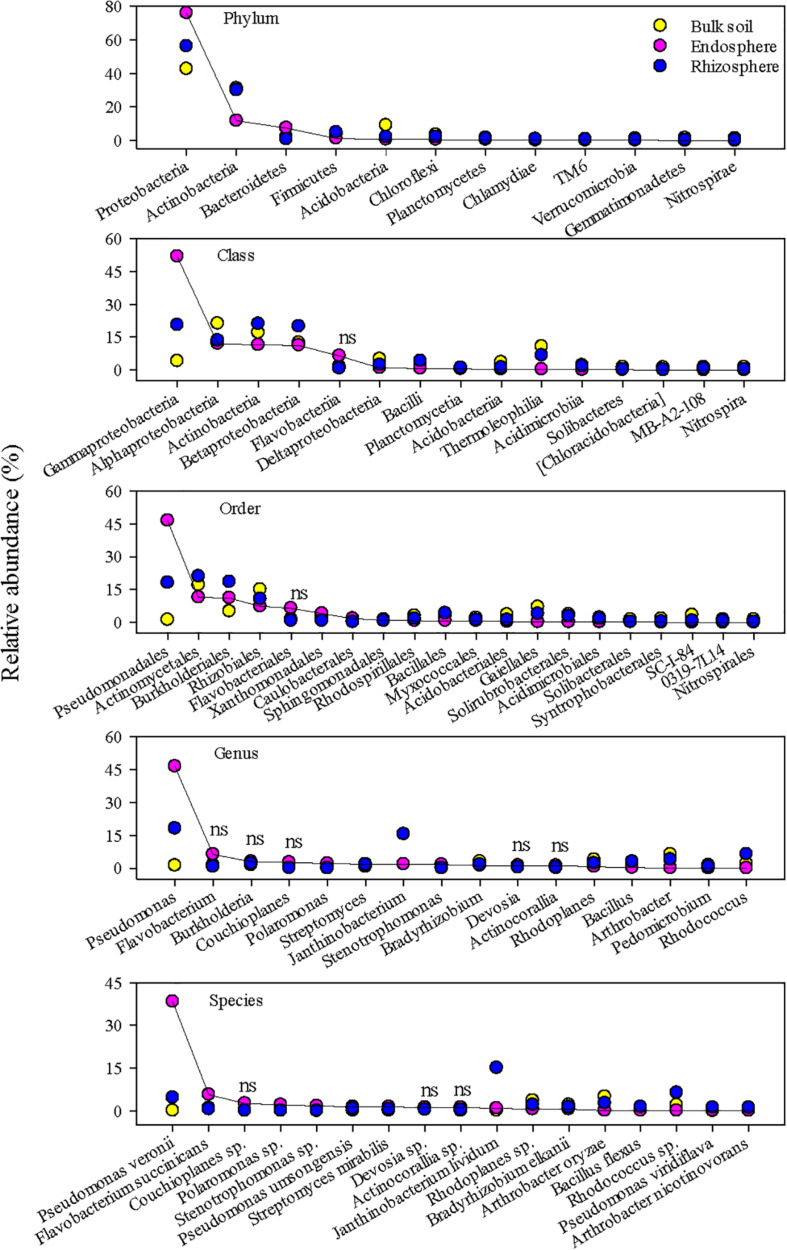
The most abundant bacterial taxa in the grass root endosphere, rhizosphere, and bulk soil. A solid line connects bacterial taxa in the endosphere to help visualize differences in relative abundance among the endosphere, rhizosphere, and bulk soil. “ns” indicates no significant difference at *P* < 0.05.

The top 20 abundant bacterial ASVs in the endosphere and rhizosphere constituted ∼58 and 41% of the respective bacterial community, compared to ∼16% in the bulk soil. The corresponding values for fungi were ∼55, 63, and 37% in the endosphere, rhizosphere, and bulk soil, respectively. Furthermore, only a few of these abundant ASVs overlapped between the three niches (Supplementary Figure 4), indicating niche-preferential taxa. Dominant bacterial taxa in the root endosphere were in sharp contrast to those in the rhizosphere and bulk soil (Figure 2). It is worth mentioning that these top bacterial taxa accounted for ∼99, 96, 93, 70, and 57% of the total abundance at the phylum, class, order, genus, and species, respectively. The endosphere enhanced the predominance of Proteobacteria, especially the class Gammaproteobacteria, accounting for ∼76 and ∼50% of the total bacterial community, respectively. Taxon discrimination of the root endosphere was also very clear at the order, genus, and species levels. Pseudomonadales and *Pseudomonas* were the most abundant order and genus in the endosphere, respectively. A single species, *P. veronii* dominated the root endosphere and constituted ∼38% on average of the total bacterial community. In contrast, the rhizosphere and bulk soil were more abundant in Alphaproteobacteria, Betaproteobacteria, Deltaproteobacteria, and Actinobacteria. *J. lividum* was the most abundant species (∼15%) in the rhizosphere whereas it only made up ∼0.1 and 0.8% in the bulk soil and endosphere, respectively. Representative 16S amplicon fragments identified as *P. veronii* and *J. lividum* are given in Supplementary Figure 5.

**FIGURE 3:**
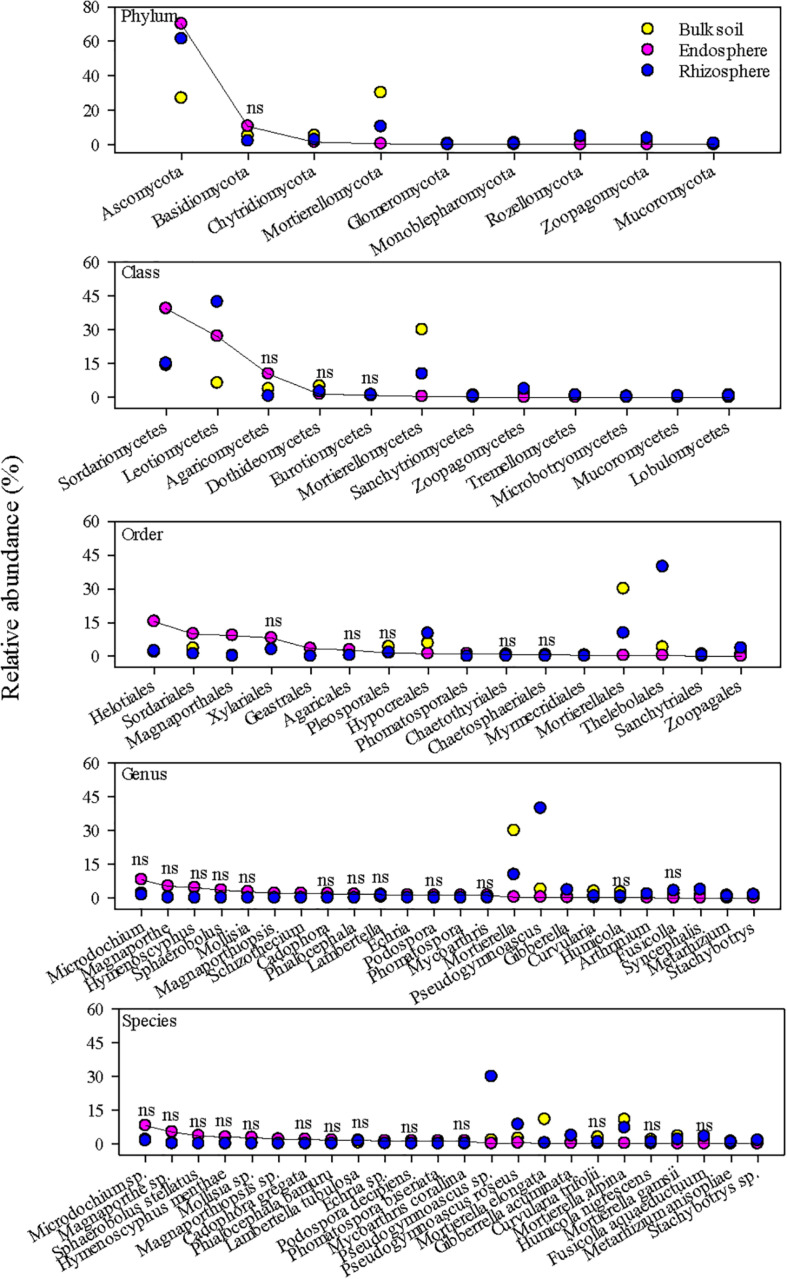
The most abundant fungal taxa in the grass root endosphere, rhizosphere, and bulk soil. A solid line connects fungal taxa in the endosphere to help visualize differences in relative abundance among the endosphere, rhizosphere, and bulk soil. “ns” indicates no significant difference at *P* < 0.05.

**FIGURE 4:**
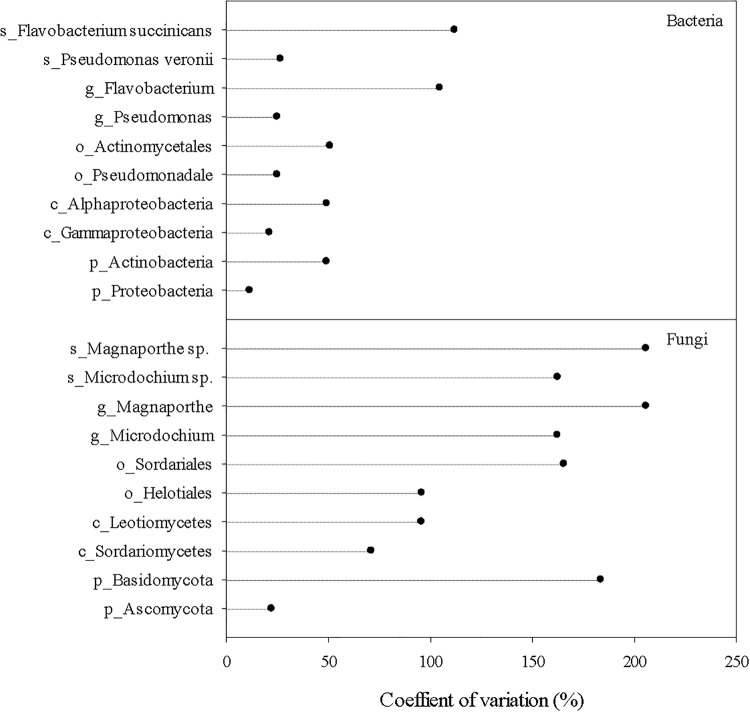
Coefficients of variation of the most abundant bacterial and fungal taxa in the root endosphere. Prefix p_, c_, o_, g_, and s_ represent phylum, class, order, genus, and species, respectively.

Dominant fungal taxa in the root endosphere also differed from those in the rhizosphere and bulk soil (Figure 3). The top fungal taxa in the root endosphere accounted for ∼83, ∼80, 55, 40, and 35% of the total abundance at the phylum, class, order, genus, and species, respectively. Ascomycota were richer in the endosphere and rhizosphere, but Mortierellomycota was much more abundant in the bulk soil. Sordariomycetes and Leotiomycetes were the major fungal classes in the endosphere, and several orders, e.g., Helotiales, Sordariales, and Magnaporthales were markedly greater in the endosphere than the bulk soil. However, endosphere-preferred taxa became less noticeable at genus and species levels. Unlike bacterial species, no fungal species in the endosphere could make up >9% of the total fungal community. In general, fungal taxa distributed more unevenly in the endosphere than bacterial taxa, as shown by larger coefficients of variation in predominant fungal genera and species than bacterial ones (Figure 4). Most predominant fungal species, i.e., species showing greater relative abundance on average, were detected in less than one third of grass root samples. For example, fungal species, *Sphaerobolus stellatus* and *Hymenoscyphus menthae* appeared in only one or two grass root samples, although these species made up ∼30–40% of the total fungal community of respective samples. In contrast, *Pseudogymnoascus* spp. were prevailing in the rhizosphere, constituting ∼39% of the total fungal community.

Bacterial and fungal distribution in the endosphere also differed in terms of their associations with turfgrass system/sampling variables as well as soil physicochemical properties. Distributions of predominant bacterial orders and genera in the endosphere were less dependent on grass species, defoliation intensity, sampling site and time, and soil physicochemical properties than fungal distributions (Supplementary Figure 6). Nonetheless, compared to the predominant bacterial and fungal species in the bulk soil, bacterial, and fungal species in the root endosphere and rhizosphere were less affected by soil physicochemical properties or turfgrass system/sampling variables (Supplementary Figure 7).

### Microbial Species Associations in the Endosphere and Rhizosphere

**FIGURE 5:**
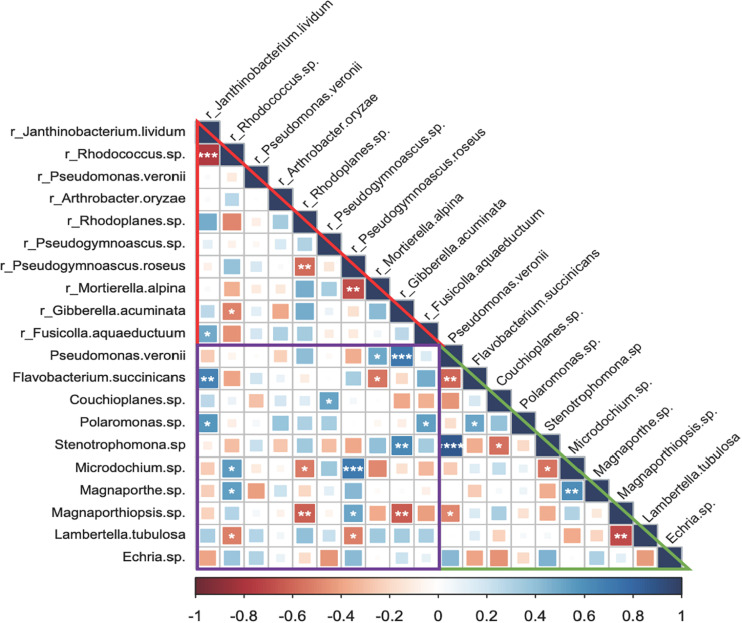
Microbial species associations (Spearman’s rank correlations) in the grass root endosphere and rhizosphere. The prefix “r_” indicates bacterial and fungal species in the rhizosphere. Triangles in red and green and a square in purple represent associations of predominant bacterial and fungal species in the rhizosphere, endosphere, and between rhizosphere and endosphere, respectively. Significant associations are marked by symbols ****, ***, **, and * at *P* < 0.001, 0.01, 0.05, and 0.1. respectively.

There were a few robust and significant species associations in the endosphere, rhizosphere, and between endosphere and rhizosphere, although most predominant bacterial and fungal species appeared to be neutrally related (Figure 5). Within the rhizosphere, associations were more negative. *J. lividum* and *Rhodococcus* sp., the two most abundant bacterial species behaved antagonistically and so did between two fungal species, *Mortierella alpine* and *Pseudogymnoascus roseus*. By contrast, both positive and negative species associations appeared in the root endosphere and between the endosphere and rhizosphere. *P. veronii*, the most abundant bacterial species in the endosphere was negatively associated with *Flavobacterium succinicans* but positively with a *Stenotrophomonas* sp. The two most abundant fungal species, *Microdochium* sp. and *Magnaporthe* sp. showed moderate but positive associations. However, fungal species *Lambertella tubulosa* and *Magnapothiopsis* sp. were negatively related. We found that *Gibberella acuminata* in the rhizosphere was correlated positively with *P. veronii* and *Stenotrophomonas* sp. and negatively with Magnoporthiopsis sp. in the endosphere. *P. roseus* in the rhizosphere positively linked to *Microdochium* sp. in the endosphere, and so did between *J. lividum* in the rhizosphere and *Flavobacterium succinicans* in the endosphere.

## Discussion

### Microbial Alpha Diversity in the Root Endosphere and Rhizosphere

As expected, both bacterial and fungal species richness was much lower in the root endosphere than species richness in the rhizosphere and bulk soil. Still, there were surprisingly hundreds of ASVs in the root endosphere, considering strong selection pressures of plant innate immune system as well as rich energy and nutrients (Kandel et al., 2017; Khare et al., 2018; Deng et al., 2019). Microbes get into grass roots through wounds and natural openings, such as root tips and lateral root emerging points. Although plants can actively recruit their microbial companions by root exudates and signaling compounds, stochastic microbial invasion into grass roots are inevitable. If microbes via stochastic invasion are not compatible with the interior environment of grass roots, they cannot proliferate and thus may exist as “rare” species. The fact that both bacterial and fungal species evenness in the root endosphere was much lower than the species evenness in the rhizosphere and bulk soil suggests a large influence of stochastic microbial invasion on species richness in the root endosphere. This is also supported by significant and positive correlations of observed or estimated bacterial ASVs between the endosphere and bulk soil and yet no significant relationship in alpha diversity metrics between the two root-associated niches, i.e., the endosphere and rhizosphere.

There was no correlation in species richness, but rather in Shannon diversity index or species evenness between the rhizosphere and bulk soil, suggesting that the rhizosphere and bulk soil shared some factors in promoting homogenous distribution of microbial taxonomic groups. However, this inference appeared not to be in line with the finding that soil properties and system variables primarily affected the bacterial and fungal diversity in the bulk soil but not in the rhizosphere or root endosphere. Physical protection (e.g., aggregation not examined in this work) is likely the factor that contributed to the nexus in diversity metrics between the rhizosphere and bulk soil. Controls on soil aggregation might vary between the bulk soil and rhizosphere (e.g., organic matter in the bulk soil versus extracellular polysaccharides in the rhizosphere), but soils are generally aggregation-tactic, favoring microbial diversity.

It is intriguing to observe marginally significant and positive correlations of soil ammonium concentration with bacterial and fungal alpha diversity metrics in the root endosphere or rhizosphere. Yet, we have no better and reasonable explanations for this phenomenon except for a possible rhizophagy process by which grass roots used diverse nutrient-rich microbes as a source of nutrients (Paungfoo-Lonhienne et al., 2010). Nonetheless, the lower diversity of bacteria and fungi in the root endosphere and rhizosphere emphasized that predominant microbial species or taxonomic groups might play a critical role in turfgrass growth and health.

### *Pseudomonas veronii* and Its Host Compatibility and Potentials in Promoting Turfgrass Health

*Pseudomonas* has been considered as the most abundant bacteria inhabiting roots of numerous plants and plays an important role in promoting the fitness of host plants by stimulating plant growth and/or suppressing pathogens (Mercado-Blanco and Bakker, 2007; Zamioudis et al., 2013; Sitaraman, 2015). As biocontrol agents and plant growth promoting rhizobacteria, for example, *Pseudomonas fluorescens*, *Pseudomonas putida*, and *Pseudomonas aeruginosa* are very popular members of the genus *Pseudomonas*. Still, more *Pseudomonas* spp. or strains are being identified to be capable of improving plant host fitness.

In this work, *P. veronii* was found to be the predominant bacterium in the root endosphere, regardless of turfgrass species, defoliation management, study sites, and sampling time, suggesting that this bacterial species was of a broad spectrum of turfgrass host compatibility. A wide array of activities by which *Pseudomonas* spp. benefit host plants have been acknowledged, including controls on phytohormones (e.g., indole acetic acid), solubilization of minerals (e.g., phosphate), and defense against plant pathogens via biocidal compounds or enhanced-plant resistance (Adhikari et al., 2001; Kuklinsky-Sobral et al., 2004; Preston, 2004; Novik et al., 2015). However, *P. veronii* has often been considered in bioremediation due to its ability of degrading a few aromatic organic compounds (Elomari et al., 1996; Nam et al., 2003). Phylogenetically, *P. veronii* is closer to *Pseudomonas fluorescence* (Anzai et al., 2000), a well-known plant growth promoting bacterium. Therefore, *P. veronii* may have potentials in promoting plant fitness to the environment. Indeed, a study on rice demonstrated that *P. veronii* not only reduced seedling diseases but also enhance plant growth in the absence of pathogens (Adhikari et al., 2001).

Genome annotation of a *P. veronii* stain that was isolated from grapevine revealed several potential traits of this species in promoting plant growth and health, including phytohormones regulation, nutrient transformations, and nematicidal activity (Montes et al., 2016). Since the relative abundance of *P. veronii* in the turfgrass root endosphere was independent of soil nitrogen, moisture, and other properties, we assumed that this species might serve as a biocontrol agent to prevent grass roots from nematode infection. Nematode issues are widespread in turfgrass systems; nematode infection on grass roots may cause a deficiency of water and nutrient uptake and therefore nematode infection may sometimes be misdiagnosed (Yu et al., 1998). Nonetheless, our turfgrass samples did not show symptoms of pathogen infection and/or unhealthy growth (personal observations), perhaps due to the contribution of *P. veronii*.

*Flavobacterium succinicans* was the second abundant bacterial species in the root endosphere, but it was negatively correlated with *P. veronii*, implying that the two bacterial species either preferred different environmental conditions or were less compatible. Given that compared to *P. veronii*, the abundance of *F. succinicans* was much lower (∼5.6%) and also varied more largely among samples, *F. succinicans* might play less roles in promoting turfgrass growth and health. Despite lower relative abundance (∼ 1.7%), a *Stenotrophomonas* sp. showed not only a strong and positive correlation with *P. veronii* but also pervasiveness in all the samples. *Stenotrophomonas* spp. are known to degrade a large range of organic compounds, including phenolics in root exudates and promote plant growth via hormone regulation and nutrient supply (e.g., nitrogen fixation and oxidation of elemental sulfur) (Ryan et al., 2009). As such, species in the genus of *Stenotrophomonas* need to be given consideration when it comes to formulate a group of bacterial species to promote turfgrass environmental fitness.

Unlike bacteria, fungi in the root endosphere varied more largely with turfgrass species, management, study site, sampling time, as well as soil physicochemical properties. The observation that these fungi appeared abundantly in a few samples and were completely absent in many others suggests that if they had any positive roles on turfgrass growth, impacts would be minor. In fact, many fungal species (e.g., *Microdochium* spp., *Magnaporthe* spp., and *L. tubulosa*) are known pathogens in turfgrass systems, although turfgrass samples were asymptomatic. As an example, *Phialocephala bamuru*, a fungal species that can cause bermudagrass fairway patch (Wong et al., 2015), was detected in an ultradwarf bermudagrass sample and two bermudagrass samples, but not in others. Hence, we considered that most abundant fungal species in the root endosphere were not plant-growth-promoting fungi.

### The Significance of *Janthinobacterium lividum* and *Pseudogymnoascus* spp. in the Turfgrass Rhizosphere

The rhizosphere contains a wide range of nutrients and is an important niche for microbial survival and activity. Microbial chemotaxis movement and subsequent competition for nutrients may result in the most adapted microbes dwelling the rhizosphere. We found several prevalent bacterial and fungal species in the turfgrass rhizosphere. The most abundant bacterial species was *J. lividum*, followed by a *Rhodococcus* sp., *P. veronii*, *Arthrobacter oryzae*, and *Rhodoplanes* sp.; and the most abundant fungal species was a *Pseudogymnoascus* sp., followed by *P. roseus* and *Mortierella alpina*. Most of these species are not pathogens and may provide some benefits to turfgrasses.

*Janthinobacterium lividum* is a Gram negative and violacein producing bacterium and possesses antifungal activity. *Janthinobacterium* in the rhizosphere of wheat displayed antagonisms against a number of soil born pathogen, including *Pythium ultimum*, *Rhizoctonia solani*, and *Rhizoctonia oryzae* (Yin et al., 2020). *Janthinobacterium* was also more abundant in the rhizosphere of asymptomatic grapevine compared to fungal pathogens-infected grapevine (Saccà et al., 2019). A recent study on the turfgrass rhizosphere also revealed that when the rhizosphere was enriched with bacterial genera *Janthinobacterium* and *Rhodococcus*, turfgrass was less susceptible to dollar spot caused by the fungal pathogen, *Clarireedia* spp. (Chou et al., 2020). Genome annotations of *Janthinobacterium* spp. further proposed nematicidal effects and also capnophilic behavior, i.e., proliferating at the presence of high concentration of carbon dioxide (Hornung et al., 2013; Valdes et al., 2015). Besides, *Janthinobacterium* was found to be able to promote plant growth by reducing plant uptake of heavy metals and producing phytohormone indole-3-acetic acid (Kuffner et al., 2008). Beneficial effects of *Janthinobacterium* appeared to be more robust at the presence of *Rhodococcus* spp. (Schmidt and Gier, 1990); authors demonstrated that *Janthinobacterium* and *Rhodococcus* together could more efficiently degrade 2,4-dinitrophenol, a major ingredient of pesticides and herbicides. Both *Janthinobacterium* and *Rhodococcus* were abundant in the turfgrass rhizosphere and yet their abundance was negatively correlated, suggesting that they responded to the rhizosphere environment differently. Given their diverse potentials in promoting plant growth, *Janthinobacterium* and *Rhodococcus* need to be further examined to determine their primary functions in turfgrass systems. *Rhodoplanes* was also the abundant bacterial genus in the rhizosphere, but its relative abundance in the rhizosphere and bulk soil was comparable. It was the same case for *A. oryzae* and *Bradyrhizobium elkanii*. This seems to contrast with the finding that *Rhodoplanes* was more enriched in the turfgrass rhizosphere (Crouch et al., 2017). To resolve this discrepancy, more data needs to be collected from turfgrasses of different species at different geographic locations.

*Pseudogymnoascus* was the most abundant fungal genus in the rhizosphere and members of this genus are usually involved in cellulose degradation (Sigler et al., 2000). *M. alpina* was also abundant in both rhizosphere and the bulk soil. They are known saprophytic fungi not only involved in the decomposition of plant debris and organic matter, but also able to benefit plant growth by enhancing apocarotenoid biosynthesis and stress tolerance (Wani et al., 2017). Thus, great abundance of *Pseudogymnoascus* and *M. alpina* in the rhizosphere may contribute to turfgrass fine root turnover and nutrient cycling and also help turfgrasses to better adapt to the environment.

## Conclusion

Turfgrasses are generally managed with supplies of optimal nutrients (e.g., nitrogen), water, and pesticides. Still, grass roots exerted strong selection pressures to recruit beneficial microbes. Several bacterial and fungal species dominated the turfgrass root endosphere and rhizosphere, including *P. veronii*, *J. lividum*, and *Pseudogymnoascus* spp. These microbes are likely involved in biocontrol, biotransformation, and plant nutrient uptake. As such, harnessing these microbial species will benefit turfgrass health and sustainability, and yet this demands a better understanding of their ecology, in particular their responses to environmental cues and interactions among these potential beneficial microbial species.

## Data Availability Statement

The datasets presented in this study can be found in online repositories. The names of the repository/repositories and accession number(s) can be found in the article/Supplementary Material.

## Author Contributions

QX contributed to data collection. QX and WS contributed to data analysis and manuscript preparation. All authors were involved in research ideal development, experimental setup, and manuscript revisions.

## Conflict of Interest

The authors declare that the research was conducted in the absence of any commercial or financial relationships that could be construed as a potential conflict of interest.
