# Supplementary material for: Predominant Microbial Colonizers in the Root Endosphere and Rhizosphere of Turfgrass Systems: Pseudomonas veronii, Janthinobacterium lividum, and Pseudogymnoascus spp
Source: Front Microbiol. 2021 Mar 23;12:643904. doi: 10.3389/fmicb.2021.643904 (PMC8021697; doi:10.3389/fmicb.2021.643904)

Supplementary Table 1. The overview of intact turfgrass-soil cores collected from different turfgrass species/cultivars, defoliation intensities, sites, and growing seasons.

| Species | Defoliation | Site [GPS]^#^ | Number – Time |
| --- | --- | --- | --- |
| Bermudagrass | Putting greens | Lake Wheeler Turfgrass Field [N35°44'21'', W78°40'68''] | Six – Nov, 2018 |
| Bermudagrass | Fairway | Lonnie Pool Golf Course [N35°45'28", W78°40'52"] | Six – Feb, 2019 |
| Bermudagrass | Fairway | NC State University Club [N35°48'05", W78°41'42"] | Six – April, 2019 |
| Ultradwarf bermudagrass | Putting greens | Lake Wheeler Turfgrass Field [N35°44'33'', W78°40'83''] | Six – Nov, 2018 |
| Ultradwarf bermudagrass | Putting greens | Lonnie Pool Golf Course [N35°45'20", W78°41'0"] | Six – Feb, 2019 |
| Ultradwarf bermudagrass | Putting greens | NC State University Club [N35°48'06", W78°41'43"] | Six – April, 2019 |
| Tall fescue | Lawn | Lake Wheeler Turfgrass Field [N35°44' 28'', W78°44'66''] | Six – Nov, 2018 |
| Tall fescue | Lawn | NC State University campus [N35°47'10", W78°40'18"] | Six – Feb, 2019 |
| Tall fescue | Lawn | A commercial plaza [N35°46'18", W78°45'34"] | Six – May, 2019 |
| Creeping bentgrass | Putting greens | Lake Wheeler Turfgrass Field [N35°44'37'', W78°40'65''] | Six – Nov, 2018 |
| Creeping bentgrass | Putting greens | NC State University Club [N35°48'05'', W78°41'42''] | Six – April, 2019 |
| Creeping bentgrass | Putting greens | Lonnie Pool Golf Course [N35°45'29", W78°40'57"] | Six – May, 2019 |

^#^GPS represents the location of the first intact turfgrass-soil core of six that were sampled at a given species and time.

Supplementary Table 2. Soil physical and chemical properties of composite samples associated with respective turfgrass species/cultivars and turf sites. See details for sampling sites and turfgrass species in Supplementary Table 1.

| Turfgrass species, site | Bulk density | Moisture | pH | NH_4_^+^-N | NO_3_^-^-N | Inorganic N | Total C | Total N |
| --- | --- | --- | --- | --- | --- | --- | --- | --- |
|  | (g cm^-3^) | (%) |  | (mg kg^-1^ soil) | | | (g kg^-1^ soil) | |
| Bermuda, Lake Wheeler | 0.99 | 18.3 | 5.8 | 5.0 | 31.9 | 36.9 | 19.4 | 1.7 |
| Bermuda, Lonnie Pool | 1.23 | 19.0 | 7.0 | 10.8 | 37.1 | 47.9 | 15.9 | 1.4 |
| Bermuda, University Club | 1.07 | 34.4 | 5.7 | 0.0 | 49.1 | 49.1 | 23.4 | 2.1 |
| Ultradwarf, Lake Wheeler | 1.35 | 4.5 | 6.8 | 22.5 | 0.6 | 23.1 | 7.2 | 0.6 |
| Ultradwarf, Lonnie Pool | 1.25 | 10.3 | 6.7 | 0.0 | 9.7 | 9,7 | 14.6 | 1.1 |
| Ultradwarf, University Club | 1.27 | 4.0 | 6.4 | 7.5 | 1.1 | 8.6 | 7.0 | 0.5 |
| Tall fescue, Lake Wheeler | 1.05 | 20.5 | 5.6 | 34.2 | 44.5 | 78.7 | 30.3 | 2.6 |
| Tall fescue, University Club | 1.19 | 22.8 | 6.4 | 0.0 | 48.7 | 48.7 | 21.3 | 1.7 |
| Tall fescue, Plaza | 1.01 | 14.3 | 6.2 | 0.0 | 9.8 | 9.8 | 26.1 | 2.0 |
| Bentgrass, Lake Wheeler | 1.37 | 7.1 | 7.5 | 10.3 | 1.3 | 11.6 | 6.1 | 0.5 |
| Bentgrass, University Club | 1.28 | 8.1 | 6.5 | 15.4 | 5.0 | 20.4 | 9.6 | 0.7 |
| Bentgrass, Lonnie Pool | 1.37 | 3.9 | 6.3 | 9.5 | 0.0 | 9,5 | 2.7 | 0.2 |

Supplementary Figure 1. (**A**) Spearman’s rho correlation coefficients of bacterial and fungal alpha diversity metrics with soil physicochemical properties. The direction and coefficient of correlation are indicated by the color and size of a square. (**B**) Kruskal-Wallis chi square statistics of bacterial and fungal diversity metrics in terms of turfgrass species/cultivars, sampling sites, defoliation intensities and sampling time. Chi-squared statistics are indicated by the size and darkness of squares. The symbol ** and * indicate a significant correlation at *P* < 0.05 and *P* < 0.1, respectively.


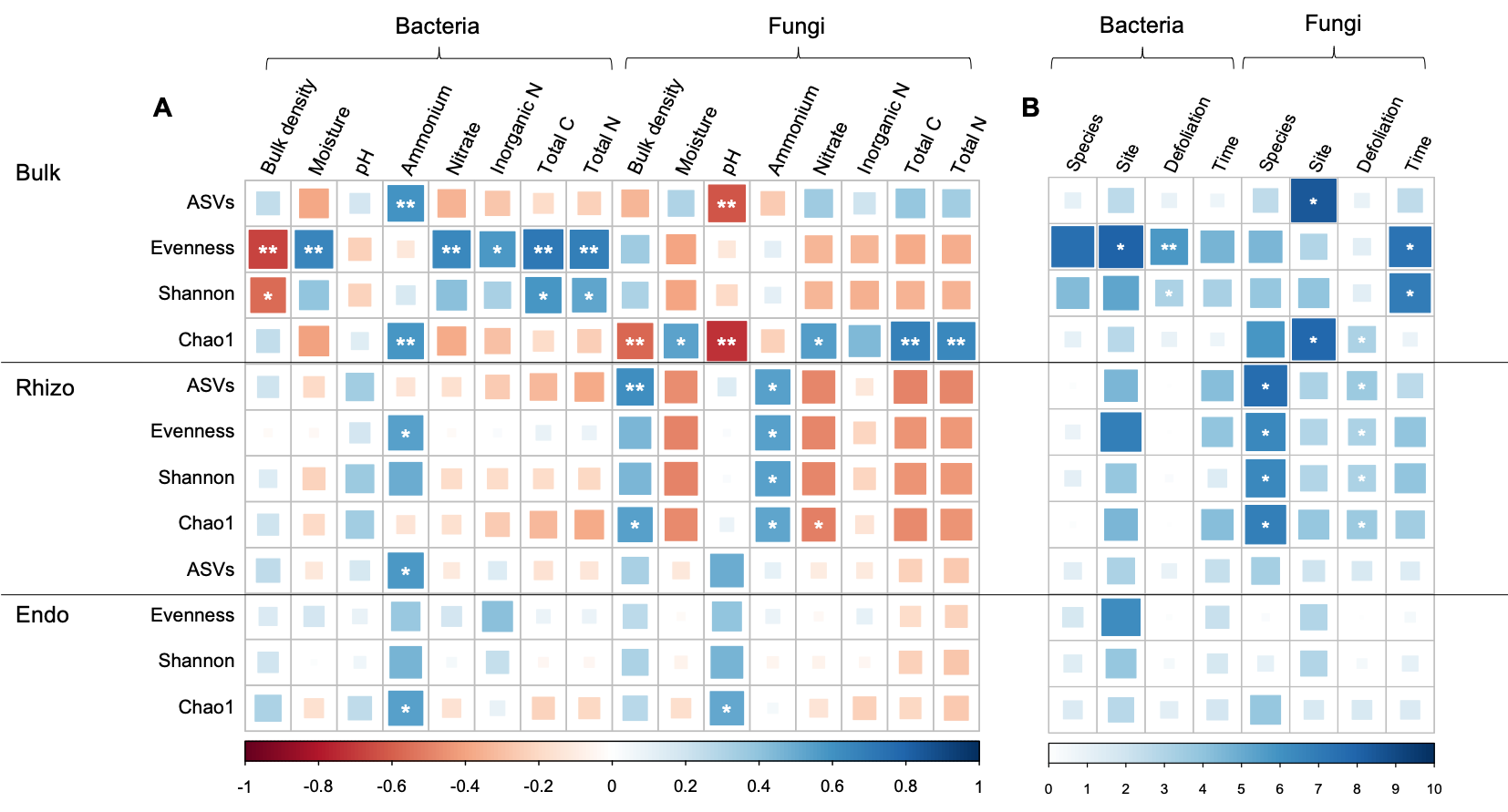


Supplementary Figure 2. (**A**) Spearman’s rho correlation coefficients of bacterial and fungal Bray-Curtis dissimilarity matrices with soil physicochemical properties. The direction and coefficient of correlation are indicated by the color and size of a square. (**B**) PERMANOVA statistics, indicated by the size and darkness of squares, of Bray-Curtis dissimilarity matrices in terms of niches (i.e., the root endosphere, rhizosphere, and bulk soil), turfgrass species/cultivars, defoliation intensity (putting greens vs. non-putting greens), sampling site and time. The symbol *** and * indicate significance at *P* < 0.001 and *P* < 0.05, respectively.


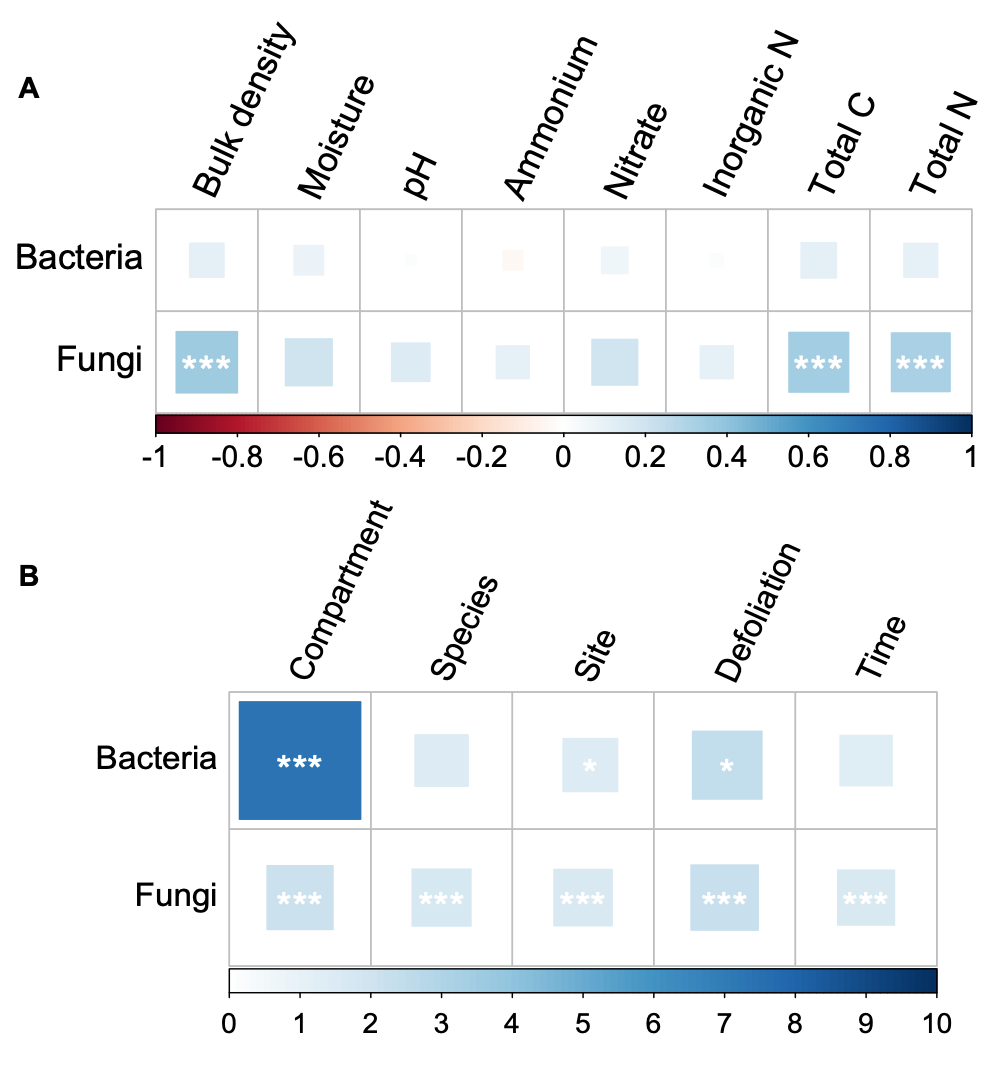


Supplementary Figure 3. Spearman’s rho correlation coefficients of bacterial (or fungal) eigenvalues along the first three PCoA axes between grass root endosphere, rhizosphere, and the bulk soil.


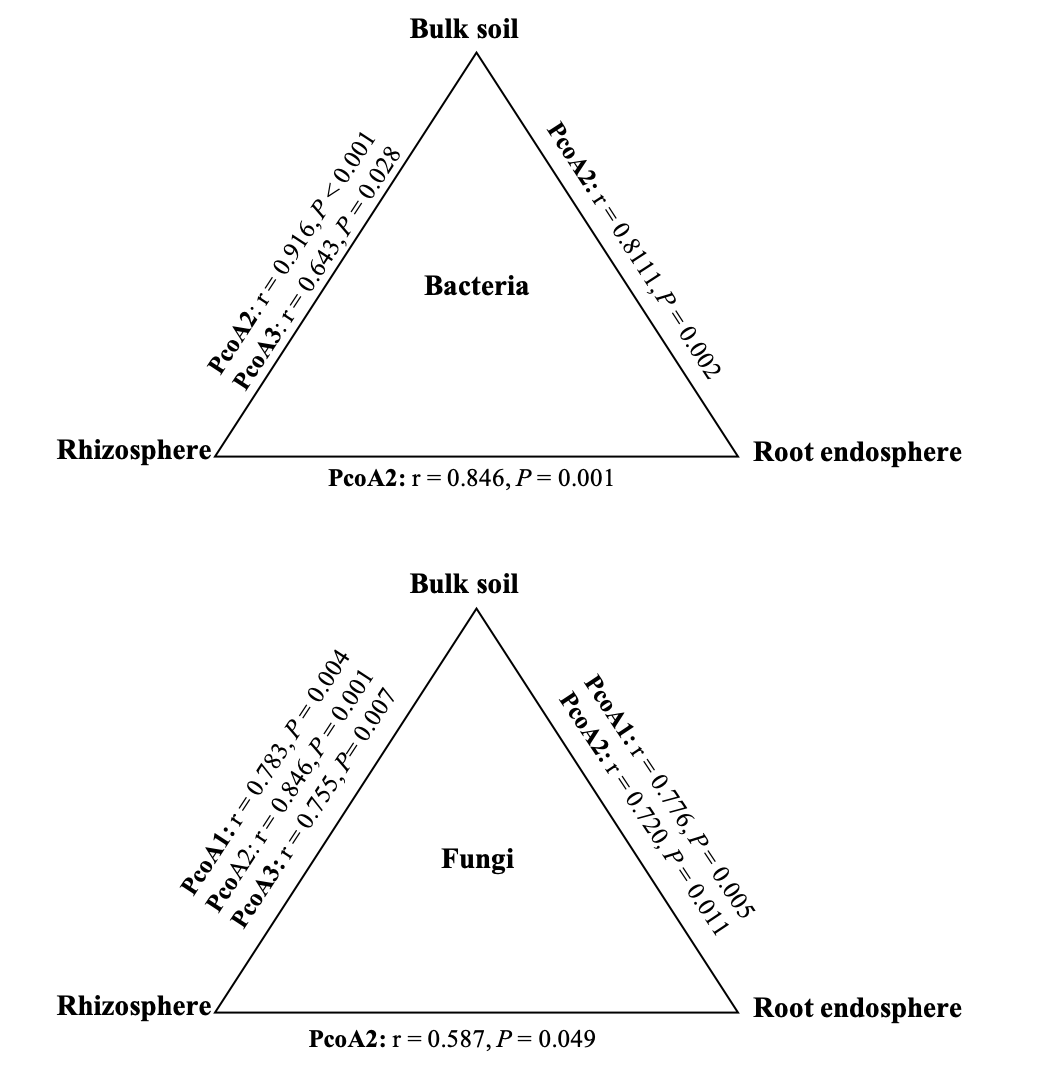


Supplementary Figure 4. A Venn diagram of the top 20 abundant ASVs in the root endosphere, rhizosphere, and bulk soil. Only three bacterial and one fungal ASVs were present in all three niches.


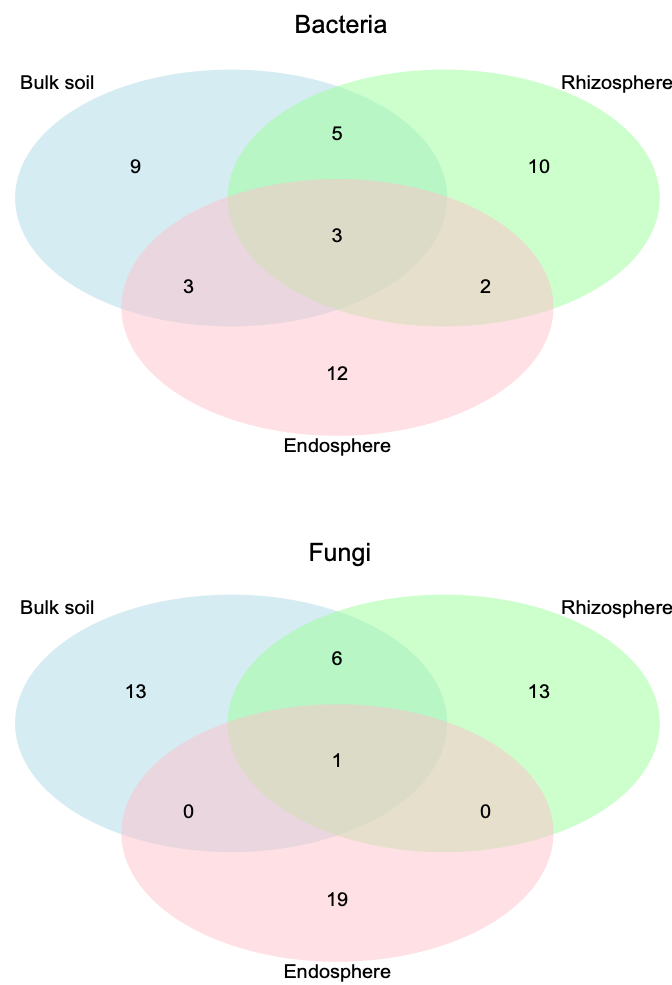


Supplementary Figure 5. Sequence alignments via Clustal Omega (www.ebi.ac.uk) of representative 16S amplicons classified as *Pseudomonas veronii* and *Janthinobacterium lividum* according to the Greengenes database. (**A**) The top three of 34 different amplicons identified as *Pseudomonas veronii* represented 87.6% (EMBOSS_001), 3.7% (EMBOSS_002), and 3.1% (EMBOSS_003) of total sequence reads. (**B**) The top three of 33 different amplicons classified as *Janthinobacterium lividum* represented 46.7% (EMBOSS_001), 21.3% (EMBOSS_002), and 13.9% (EMBOSS_003) of total sequence reads.
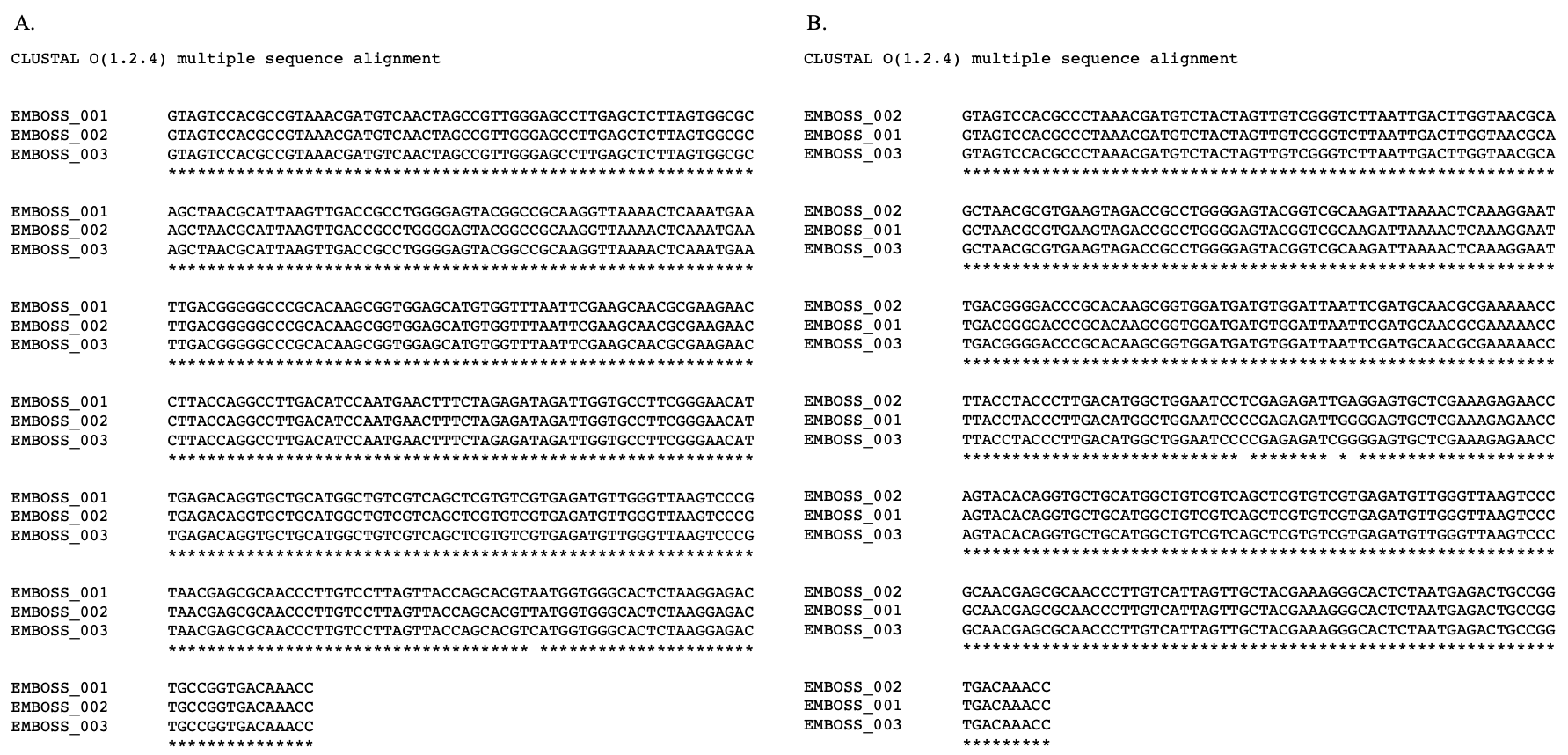
 o

Supplementary Figure 6. Statistical overview of most abundant bacterial and fungal orders and genera in grass roots. (**A**) and (**C**) are Kruskal-Wallis statistics for bacteria and fungi, respectively. (**B**) and (**D**) are Spearman’s rank correlation coefficients for bacteria and fungi, respectively. Symbols ***, ** and * represent the significance at *P* < 0.001, 0.01, and 0.05, respectively.


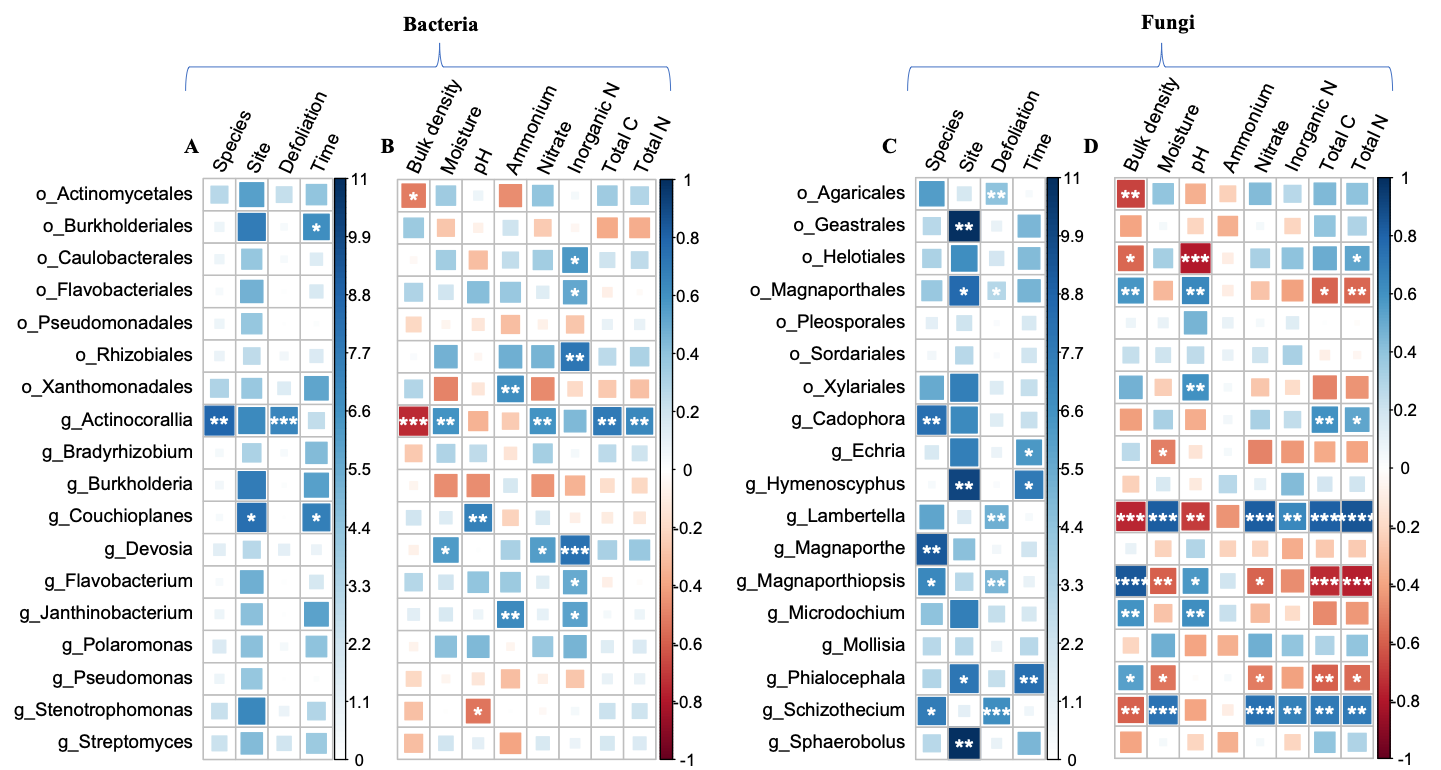


Supplementary Figure 7. Statistical overview of the five most abundant bacterial and fungal species in the bulk soil (Bulk), rhizosphere (Rhizo), and root endosphere (Endo). (**A**) and (**C**) are Kruskal-Wallis statistics for bacteria and fungi, respectively. (**B**) and (**D**) are Spearman’s rank correlation coefficients for bacteria and fungi, respectively. Symbols ***, ** and * represent the significance at *P* < 0.001, 0.01, and 0.05, respectively.


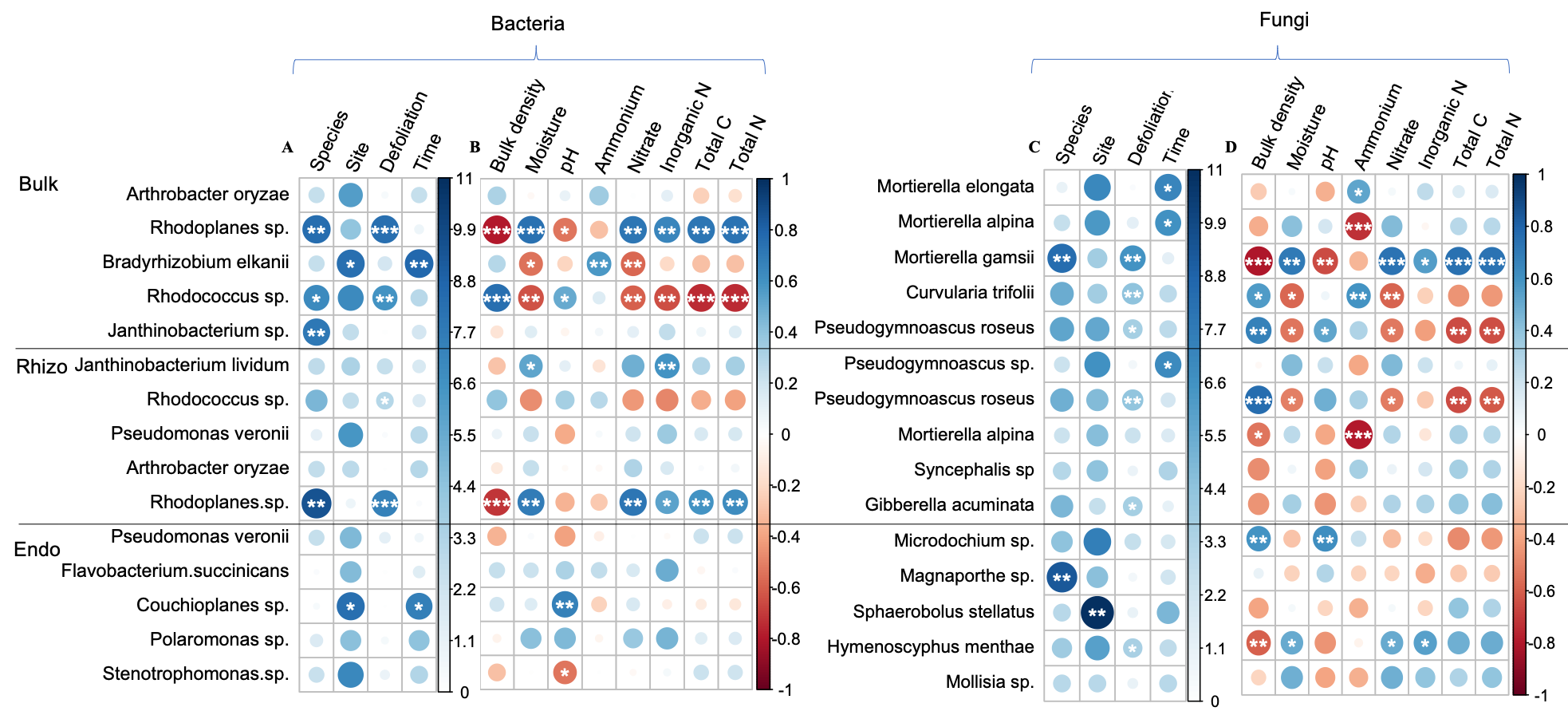

Supplement: Supplementary file 1 [file Data_Sheet_1.docx]
